# Characterization of Isoenzyme-Selective Inhibitors of Human Sphingosine Kinases

**DOI:** 10.1371/journal.pone.0044543

**Published:** 2012-09-10

**Authors:** Peng Gao, Yuri K. Peterson, Ryan A. Smith, Charles D. Smith

**Affiliations:** 1 Department of Drug Discovery and Biomedical Sciences, Medical University of South Carolina, Charleston, South Carolina, United States of America; 2 Apogee Biotechnology Corporation, Hummelstown, Pennsylvania, United States of America; Virginia Commonwealth University School of Medicine, United States of America

## Abstract

Sphingosine kinases (SKs) are promising new therapeutic targets for cancer because they regulate the balance between pro-apoptotic ceramides and mitogenic sphingosine-1-phosphate. The functions of the two SK isoenzymes, SK1 and SK2, are not redundant, with genetic ablation of SK2 having more pronounced anticancer effects than removal of SK1. Although several small molecule inhibitors of SKs have been described in the literature, detailed characterization of their molecular and cellular pharmacology, particularly their activities against human SK1 and SK2, have not been completed. Computational modeling of the putative active sites of SK1 and SK2 suggests structural differences that might allow isozyme-selective inhibitors. Therefore, we characterized several SK-inhibitory compounds which revealed differential inhibitory effects on SK1 and SK2 as follows: SKI-II and ABC294735 are SK1/2-dual inhibitors; CB5468139 is a SK1-selective inhibitor; and ABC294640 is a SK2-selective inhibitor. We examined the effects of the SK inhibitors on several biochemical and phenotypic processes in A498 kidney adenocarcinoma cells. The SK2-selective inhibitor ABC294640 demonstrated the most pronounced effects on SK1 and SK2 mRNA expression, decrease of S1P levels, elevation of ceramide levels, cell cycle arrest, and inhibition of proliferation, migration and invasion. ABC294640 also down-regulated the expression or activation of several signaling proteins, including STAT3, AKT, ERK, p21, p53 and FAK. These effects were equivalent or superior to responses to the SK1/2-dual inhibitors. Overall, these results suggest that inhibition of SK2 results in stronger anticancer effects than does inhibition of SK1 or both SK1 and SK2.

## Introduction

Sphingosine kinases (SKs) catalyze the phosphorylation of sphingosine to generate sphingosine-1-phosphate (S1P). Ceramide and sphingosine, which are upstream of SKs, are pro-apoptotic [1], [2], while S1P promotes proliferation, inflammation and migration [3], [4]. Therefore, SKs balance the levels of S1P and ceramide, and so are being increasingly recognized as potential targets for anticancer drugs [5], [6]. However, because two SK isoenzymes exist [7], [8], it is important to determine if SK1, SK2 or both should be targeted for cancer chemotherapy.

The SKs are encoded by distinct genes with 45% identity and 80% similarity in their amino acid sequences, and share five conserved domains [8]. Although no crystal structure is available, the SKs share homology with the catalytic domain of diacylglycerol (DAG) kinase [9], for which a crystal structure has been published [10]. Several topologic and functional differences between SK1 and SK2 have been described. For example, SK1 is a cytosolic protein that migrates to the plasma membrane upon activation by several stimuli [11]. Up- and down-regulation of SK1 expression results in pro- and anti-cancer effects, respectively [12], [13]. Conversely, SK2 contains a nuclear localization signal, which results in both nuclear and cytosolic protein when overexpressed [14]. The role of SK2 in cell proliferation has been somewhat unclear. On one hand, SK2 contains a pro-apoptotic BH3 domain which promotes apoptosis when this protein is over-expressed [15]. Alternately, down-regulation of SK2 inhibits the proliferation of tumor cells [16], [17], and the growth of SK2-deficient xenografts in mice is significantly delayed [18].

Although several small molecule inhibitors of SKs have been described, detailed characterizations of their pharmacology, particularly their selectivity against human SK1 and SK2, have not been completed. The first known SK inhibitors were sphingosine analogues such as N,N-dimethyl-D-erythro-sphingosine (DMS) that block the activities of both SK1 and SK2 by competing with the natural substrate sphingosine [19], [20]. DMS is reported to inhibit tumor growth and to induce cancer cell apoptosis [21]–[23]; however, DMS also inhibits PKC and other kinases, and therefore is not considered to be an SK-specific inhibitor [24], [25]. A few compounds have been described as SK1-selective inhibitors, including SK1-I which reduces the growth rate of glioblastoma and AML xenografts [26], [27], and SKI-178 which inhibits the proliferation of a variety of cancer cell lines [28]. However, these compounds are not commercially available or lack of characterization *in vivo* [29], [30]. We reported that SKI-II can inhibit SK1, and that it reduces S1P production in mouse mammary adenocarcinoma cells [31], [32]. This compound has been widely used as a SK1 inhibitor; however, we show now that it is active against both SK1 and SK2. ABC294640 is an SK2-selective inhibitor that has antitumor activity *in vitro* and *in vivo* [33], [34], and is currently in phase I clinical testing. Finally, SG14 is reported to specifically inhibit SK2 without affecting PKC [35].

To provide a more complete characterization of SK inhibitors, we herein determine the pharmacologic properties of a panel of previously reported SK inhibitors, as well as a new SK1-selective inhibitor, and compare their effects on A498 kidney adenocarcinoma cells. Our results suggest that SK2-selective inhibitors may have better antitumor activity than SK1-selective or SK1/2-dual inhibitors.

## Materials and Methods

### Cell Lines and Reagents

**Figure 1:**
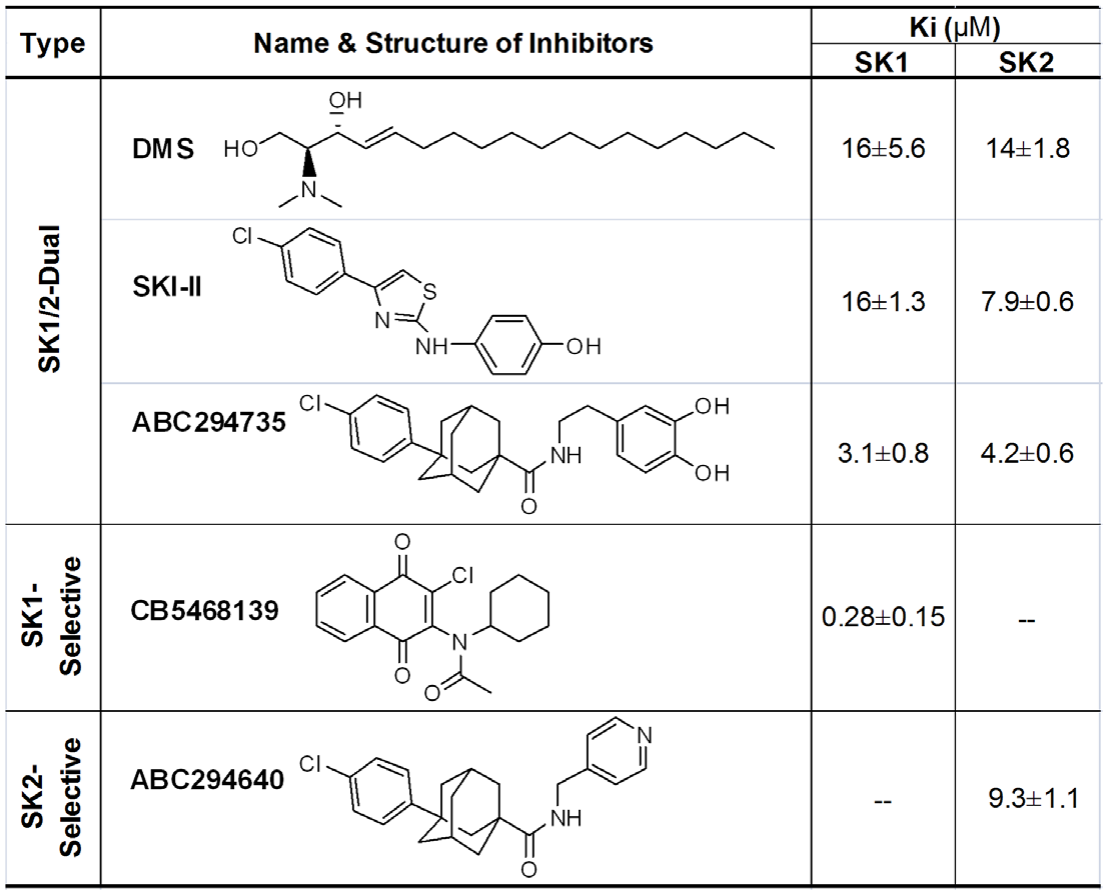
Structures and potencies of SK inhibitors. Recombinant human SK1 and SK2 were assayed in the presence of varying concentrations of the indicated compounds, and K_i_s were calculated as described in the Materials and Methods section. Data are mean ± SEM of three independent experiments.

A498 kidney adenocarcinoma cells were from the American Type Culture Collection (purchased in 2011 and 2007, ATCC authentication by isoenzyme analysis and STR analysis) and cultured in MEM contiaining 10% FBS and 50 µg/ml gentamicin. ABC294640 and ABC294735 (Figure 1) were synthesized as described previously [36], CB5468139 was from ChemBridge Corporation (San Diego, CA), and all other chemicals were from Sigma-Aldrich.

### SK Activity Assays

The enzymatic activities of recombinant human SK1 or SK2 (purity ≥74% by coomassie staining for each isozyme, BPS Biosciences) were measured using the ADP-Quest kinase assay kit (DiscoveRX Corporation, Fremont, CA) as previously described [34]. To determine the K_m_ for sphingosine, SK1 or SK2 assays were conducted using 100 µM ATP and sphingosine varying from 1.25 to 20 µM. The reactions were maintained in initial velocity conditions (<5% of the substrate consumed), and data was fitted to the Michaelis–Menten equation by nonlinear regression (Prism 5 for Windows, GraphPad Software). In determining the effects of the SK inhibitors, sphingosine was used at the K_m_ for each isoenzyme (10 and 5 µM for SK1 and SK2, respectively), the test compound was dissolved in DMSO and the ADP-Quest assay was conducted. Because SKI-II was found to interfere with this assay, we used the florescence-based HPLC assay we described previously [34] to characterize this compound. For substrate competition analyses, a series of sphingosine concentrations were used and Lineweaver–Burk plots were constructed to define the mode of inhibition.

### Homology Modeling and Docking Simulations

Modeling, simulations and visualizations were performed using MOE Version 2009.10 (Chemical Computing Group). Multiple sequence alignment was performed using BioEdit v7.0.9 [37]. The human amino acid sequences for SK1 and 2 were aligned individually with the sequence of the x-ray crystal structure of *Staphylococcus aureus* DAG kinase (PDB 2QV7). The kinase domain of SK is recognized by the NCBI conserved domains database as a DAG kinase domain (COG1597: LCB5). For computational docking of S1P to SK1 and SK2, homology models with ADP bound were generated using PDB accession code 2QV7 as the input. Prior to the analyses and simulations, the DAG kinase protein was protonated at pH 7.5 and the structure was energy-minimized. Two-phased docking consisted of a primary dock calculating 50 poses using triangle matcher placement and London dG scoring. The top 30 poses for each compound were refined using forcefield placement and Affinity dG scoring.

### Phenotypic Assays

To assess proliferation, cells were treated with the SK inhibitors for the indicated times, and cell numbers were quantified using the sulforhodamine assay [38]. Expression levels of SK1 and SK2 were measured by quantitative PCR using GAPDH for normalization, as previously described [17]. For sphingolipid mass measurements, cells were treated with inhibitors at their respective IC_50_ for 48 hr, and then washed with PBS. The cell pellets were subjected to sphingolipid profiling by HPLC-MS by the Lipidomics Core Facility at MUSC as described elsewhere [34]. In the migration and invasion assays, cells were harvested after treatment with inhibitors at their respective IC_50_ for 48 hr, washed and suspended in serum-free media with the same concentration of the inhibitor. For migration assays, 50,000 cells were placed in each cell culture insert (24-well format, 8 µm pore size, BD Biosciences) for 1 hr at room temperature. For invasion assays, matrigel pre-coated inserts were used as previously described [17]. In both cases, inserts were placed into 24-well plates with 0.3 ml of 10%-serum-containing medium having the same concentration of the inhibitor as the insert. Cells were cultured for 4 hr for migration or 24 hr for invasion assays, and the number of cells that migrated to the underside of the inserts was determined by crystal violet staining as previously described [17].

### Western Blot Analyses

Following treatment with the SK inhibitors, cells were washed, harvested and lysed, and supernatants were prepared and protein concentrations were determined as previously described [17]. Immunoblotting was carried out with the following primary antibodies (Cell Signaling): AKT, pAKT, ERK1/2, pERK1/2, STAT3, pSTAT3, p21, p53, FAK, pFAK(Y397), Beclin1, LC3, β-actin; and corresponding HRP-conjugated anti-rabbit or anti-mouse secondary antibodies, and immunocomplexes were visualized by the chemiluminescence. Protein expression was quantified by densitometric scanning of the BIOMAX XAR films after normalization to β-actin using the program NIH ImageJ.

## Results

### Molecular Pharmacology of SK Inhibitors

#### SK kinetic parameters

**Figure 2:**
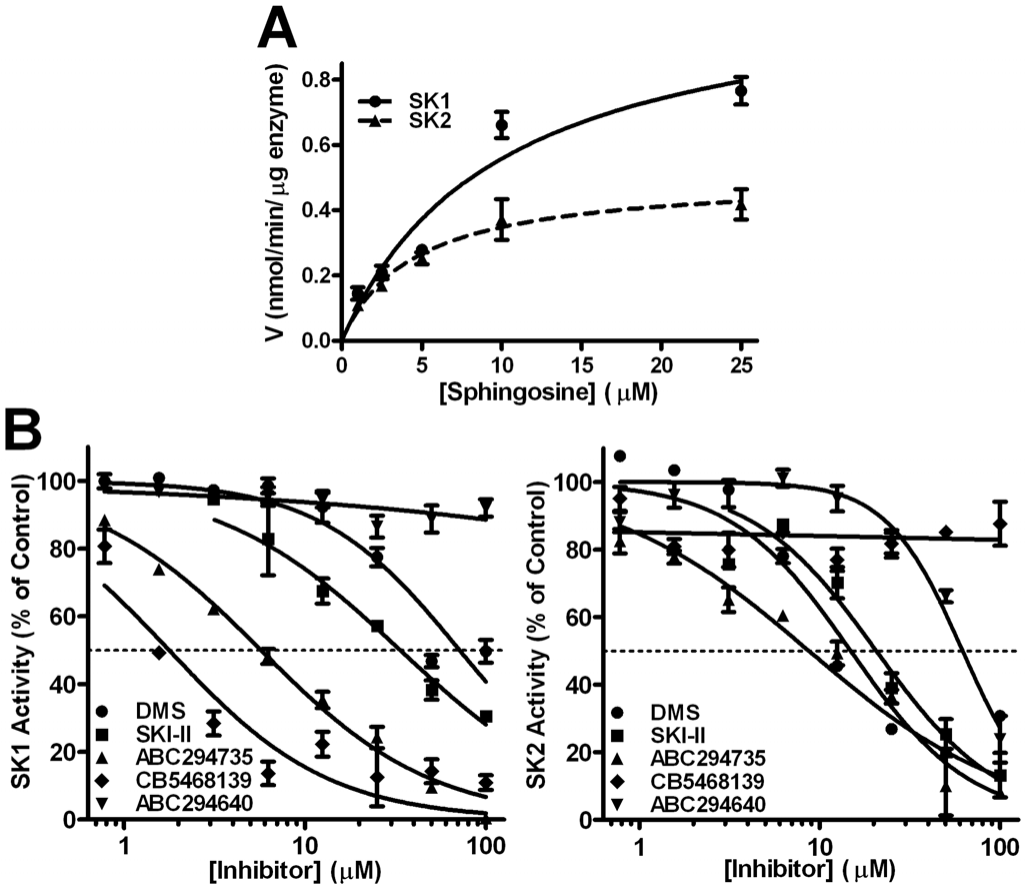
SK kinetics and inhibition. *A)* The activities of SK1 (•) and SK2 (▴) were measured under initial velocity conditions at the indicated concentrations of sphingosine as described in the Materials and Methods section. *B)* SK1 (left panel) and SK2 (right panel) activities were measured in the presence of the indicated concentrations of DMS (•), ABC294735 (▴), CB5468139 (⧫) or ABC294640 (▾) using the ADP Quest assay or SKI-II (▪) using the HPLC assay as described in the Materials and Methods section. Data are mean ± SD of triplicates of a representative of three independent experiments.

It was first necessary to determine the Michaelis-Menten constant (K_m_) for sphingosine for recombinant human SK1 and SK2. Varying the sphingosine concentration under initial velocity assay conditions provided K_m_s of 10±0.45 µM and 5±0.36 µM for SK1 and SK2, respectively. The maximal rate (V_max_) catalyzed by SK1 was approximately double that of SK2 (Figure 2A), suggesting a higher catalytic turnover number for SK1.

#### SK inhibition

Five small molecule inhibitors (Figure 1) were evaluated for their inhibitory effects and selectivity toward recombinant SK1 and SK2 using sphingosine concentrations of 10 and 5 µM, respectively. Dose-response curves for inhibition of SK1 and SK2 are shown in Figure 2B and indicate that DMS inhibits SK1 and SK2 with similar potencies (IC_50_∼60 and 20 µM, respectively). SKI-II demonstrated slightly higher potency toward SK2 (IC_50_ = 20 µM) than toward SK1 (IC_50_ = 35 µM). This compound has been widely cited as an SK1 inhibitor, but these data indicate that it is a SK1/2-dual inhibitor which favors SK2 inhibition. We have previously described phenyladamantane-based compounds that inhibit SKs [34], [36], [39]. ABC294735 is one of the most potent compounds in this series causes dose-dependent inhibition of both SK1 and SK2 (IC_50_s∼10 µM), and is therefore considered to be a SK1/2-dual inhibitor. ABC294640 demonstrates dose-dependent inhibition on SK2 with an IC_50_∼40 µM without impacting the activity of SK1 at concentrations up to at least 100 µM consistent with our previous report [34]. CB5468139 was identified by screening the ChemBridge Corporation Diverset collection for SK1 inhibitors and caused dose-dependent inhibition of SK1, with a high potency (IC_50_∼2 µM) without affecting SK2 activity up to at least 100 µM. Therefore, we now report CB5468139 to be a commercially-available SK1-selective inhibitor.

**Figure 3:**
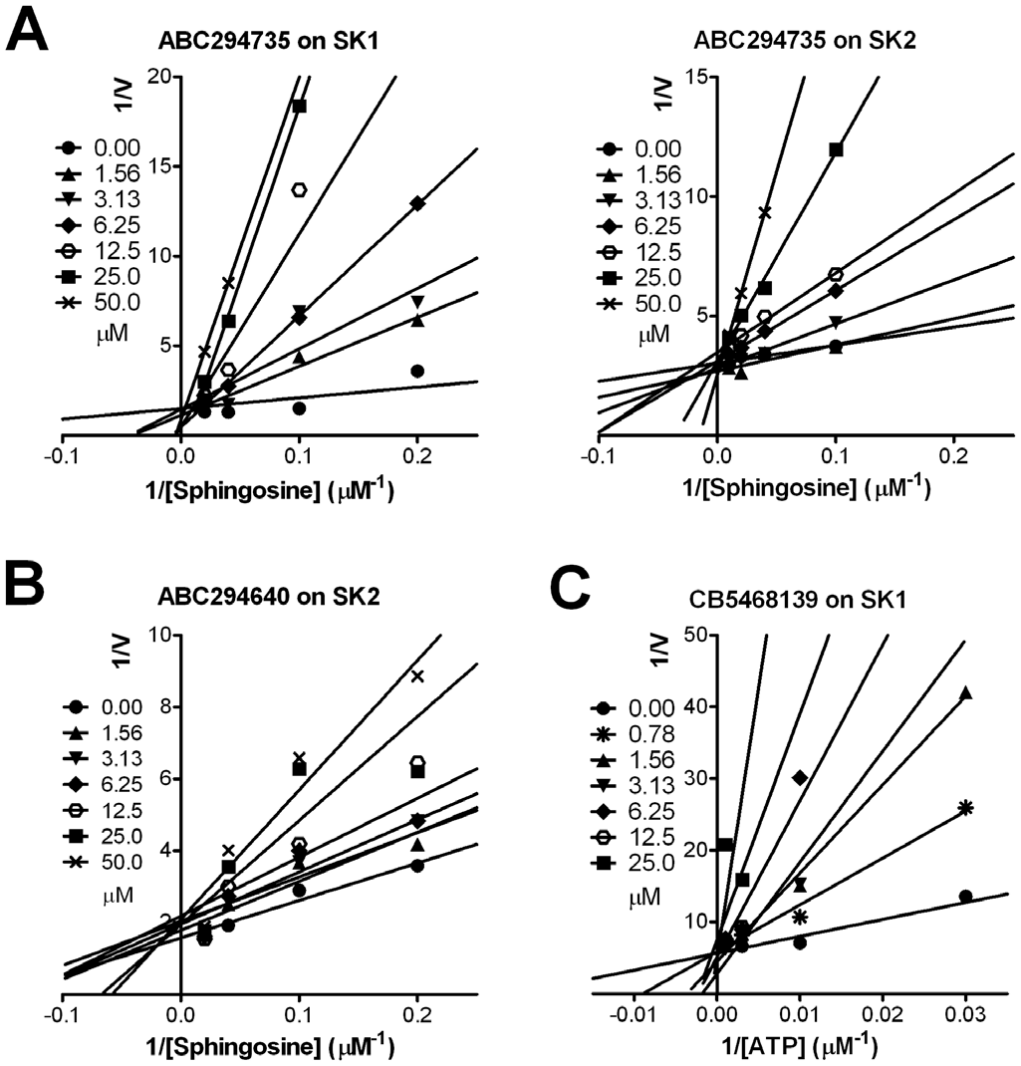
Substrate competition by SK inhibitors. SK1 (Panels A left and C) or SK2 (Panels A right and B) were assayed in the presence of the indicated concentrations of sphingosine and 0 (•), 1.56 (▴), 3.13 (▾), 6.25 (⧫), 12.5 (○), 25 (▪) or 50 (**X**) µM ABC294735 (Panel A) or ABC294640 (Panel B); or 0 (•), 0.78 (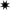), 1.56 (▴), 3.13 (▾), 6.25 (⧫), 12.5 (○) or 25 (▪) µM CB5468139 (Panel C). Data are representative of at least 2 independent experiments for each compound.

To further investigate the inhibitory mechanisms of the SK inhibitors, we conducted substrate competition assays with sphingosine or ATP. As expected [23], DMS demonstrated competitive inhibition with respect to sphingosine (data not shown). Lineweaver-Burk plots demonstrate that ABC294735 is a sphingosine-competitive inhibitor for both SK1 and SK2 (Figure 3A), and that the SK2-selective compound, ABC294640, is also a sphingosine-competitive inhibitor (Figure 3B). Because of this, the IC_50_ values depend on the concentration of sphingosine used in the assays and so K_i_s for each compound were calculated as a more appropriate means for comparing their inhibitory potencies. As tabulated in Figure 1, DMS is the least potent dual-inhibitor with K_i_s of 16 and 14 µM for SK1 and SK2, respectively, which are consistent with previous reports [8], [40]. SKI-II and ABC294735 have similar K_i_s towards SK1 and SK2, with ABC294735 being substantially more potent than SKI-II. Although ABC294640 (K_i_ = 9.3 µM) is not as potent as ABC294735, its SK2-selectivity and pharmacological properties *in vivo* made it a superior clinical candidate [34]. From its structure, CB5468139 was suspected to be an ATP mimetic, and this was confirmed through Lineweaver-Burk analyses (Figure 3C). The targeting of the ATP binding site by CB5468139 was confirmed by profiling its effect on a panel of protein kinases, which demonstrated that 2 µM resulted in ≥50% inhibition of 12 of 65 protein kinases tested (Figure S1). Nevertheless, the fact that CB5468139 is much more potent toward SK1 than SK2 indicates that the catalytic sites of the two isoenzymes are sufficiently different to allow selective pharmacologic inhibition. Because ABC294640 is selective toward SK2 it appears that the sphingosine binding site also significantly differs between SK1 and SK2. Therefore, we sought structural insight to these selective binding properties through computational modeling of the catalytic domains of the SK isoenzymes.

### Computational Chemistry

#### Homology modeling

Presently, there is no experimental structural information available for either SK1 or SK2. However, BLAST comparisons of the SK sequences against the PDB database indicted that DAG kinase is highly similar in the catalytic domain [8]. Although a homology model of SK1 was described by Kennedy [29], there is no similar homology model for SK2 or side by side comparison of SK1 and SK2. Pairwise and multiple alignments show a very good degree of similarity among DAG kinase, SK1 and SK2 (Figure S2). Alignment of DAG kinase with SK1 indicated 83 identities and 79 similarities, giving an overall similarity of 46%, while alignment of DAG kinase to SK2 indicated 74 identities and 85 similarities, and if the SK2 insertion is ignored for statistical comparison, an overall similarity of 51%. Because the x-ray crystal structure of DAG kinase has been solved at a 2.8 Å [10], we used this as a template to build homology models for human SK1 and SK2.

**Figure 4:**
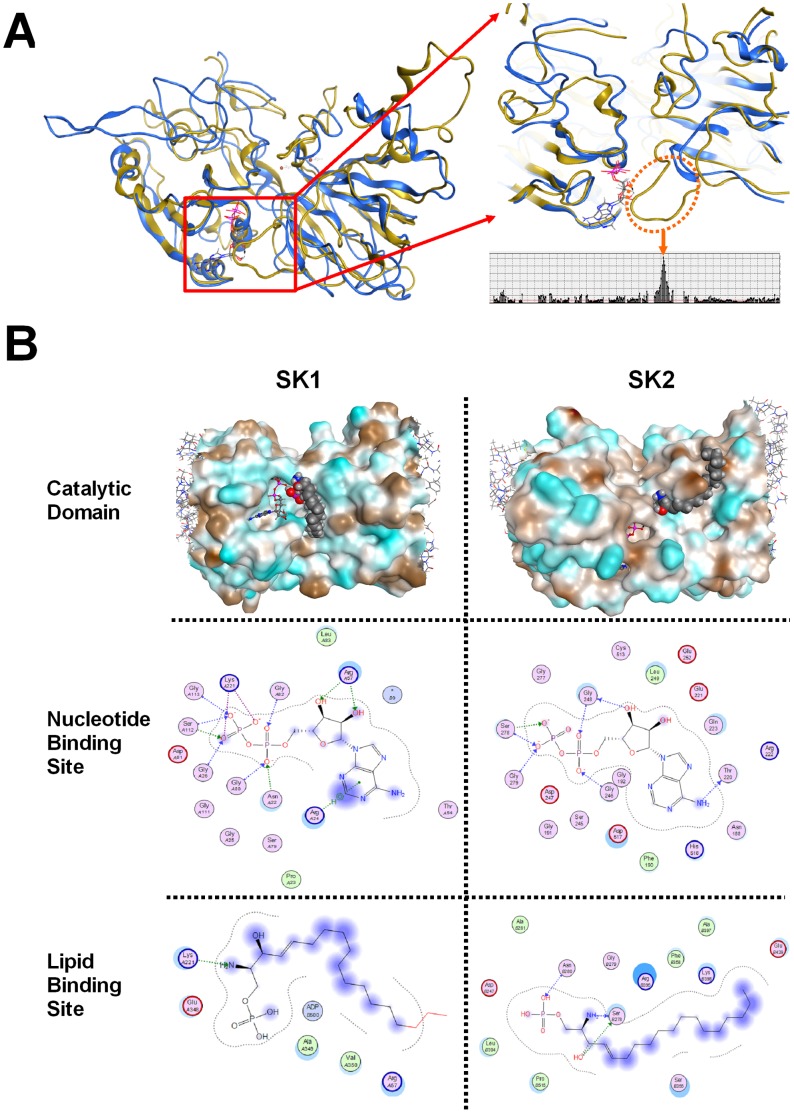
Structural comparison of SK1 and SK2. Homology models for the catalytic domain of human SK1 and SK2 were created as described in the Materials and Methods section. ***A*** *)* The ribbon model for SK1 is shown in blue and SK2 in yellow. The expanded section highlights the extended loop found in the lipid binding domain of SK2, and bars indicate the distance between residues of SK1 and SK2. ***B*** *)* The nucleotide and lipid binding domains of SK1 (left) and SK2 (right) were compared. Top Row: residues in the surface images of the catalytic domain are colored brown for hydrophobic and blue for hydrophilic. ADP is indicated by the ball-and-stick model, and S1P by the space-filling model. Middle Row: interaction diagrams of ADP binding to SK. Bottom Row: interaction diagrams of S1P binding to SK.

The primary goal of the modeling and simulation studies was to better understand the topology and chemistry of the SK active sites, not to predict the overall structure of the full-length enzymes. Comparison of the homology models of SK1 and SK2 (Figure 4A) revealed that the overall RMSD divergence of the two models was 4.96 Å which is due to several insertions and deletions in the SK1 and SK2 sequences. Working from the amino-terminus of the model (numbered residues 176–666 in Figure S2), there are a series of five short inserts in both SKs that are not present in DAG kinase. Additionally, SK2 contains a large insertion (numbered residues 391–481 in Figure S2) located directly proximal to the lipid binding domain. This results in a large loop which may restrict access to the catalytic site of SK2 (Figure 4A), possibly resulting in the decreased catalytic efficiency of the enzyme compared with SK1. Nonetheless, the overall structure and lipophilicity (Figure 4B) of the catalytic domains of SK1 and SK2 are predicted by these models to be very similar.

Considering the predicted structural similarities between SKs and DAG kinase, we determined the effects of the SK inhibitors on DAG kinase activity using the same assay as for SK. The IC_50_s for inhibition of DAG kinase by ABC294640 or CB5468139 were well above 100 µM Figure S3). The lack of inhibitory activity of CB5468139 is possibly due to the unique ATP binding motif of DAG kinase [41]. While DMS was a weak inhibitor of DAG kinase with an IC_50_ of ∼110 µM, SKI-II and ABC294735 were potent inhibitors with IC_50_s of approximately 2 and 7 µM, respectively. We also tested the effects of DAG on SK1 and SK2 activity, and found no inhibition of either isozyme up to at least 100 µM (data not shown).

#### Substrate binding

To further understand the catalytic mechanism of the SK isoenzymes, S1P was docked to SK1 and SK2 containing bound ADP (Figure 4B) with emphasis on interaction poses where the phosphate headgroup of S1P was in close proximity to beta phosphate of ADP. For both SK1 and SK2, the predicted nucleotide binding pocket interactions are similar to those of other kinases, with several glycines donating protons to the charged oxygens of the alpha and beta phosphates of ADP. The beta phosphate also appears to interact with a serine residue, and a threonine residue accepts a primary amine proton from the nucleotide base in both models.

In contrast to nucleotide binding, the predicted sphingosine binding interactions were very dissimilar between the SK1 and SK2 models. In SK1, Lys221 donates a side-chain proton to the amine nitrogen of S1P, and an oxygen from the beta phosphate of ADP forms a hydrogen bond with the S1P headgroup. In SK2, bonding is predicted between side-chain atoms from Asn280 and the phosphate headgroup of S1P, as well as between Ser278 and the S1P amino and hydroxyl groups. This SK2 model suggests that conformational rearrangements facilitate substrate binding and product release. Unexpectedly in the SK2 model, the interaction of the alkene moiety of S1P does not appear to be primarily based on hydrophobic interactions because the lipid lies in a relatively neutral groove tangential to the hydrophilic nucleotide binding cavity.

### Cellular Pharmacology of SK Inhibitors

#### Proliferation

**Figure 5:**
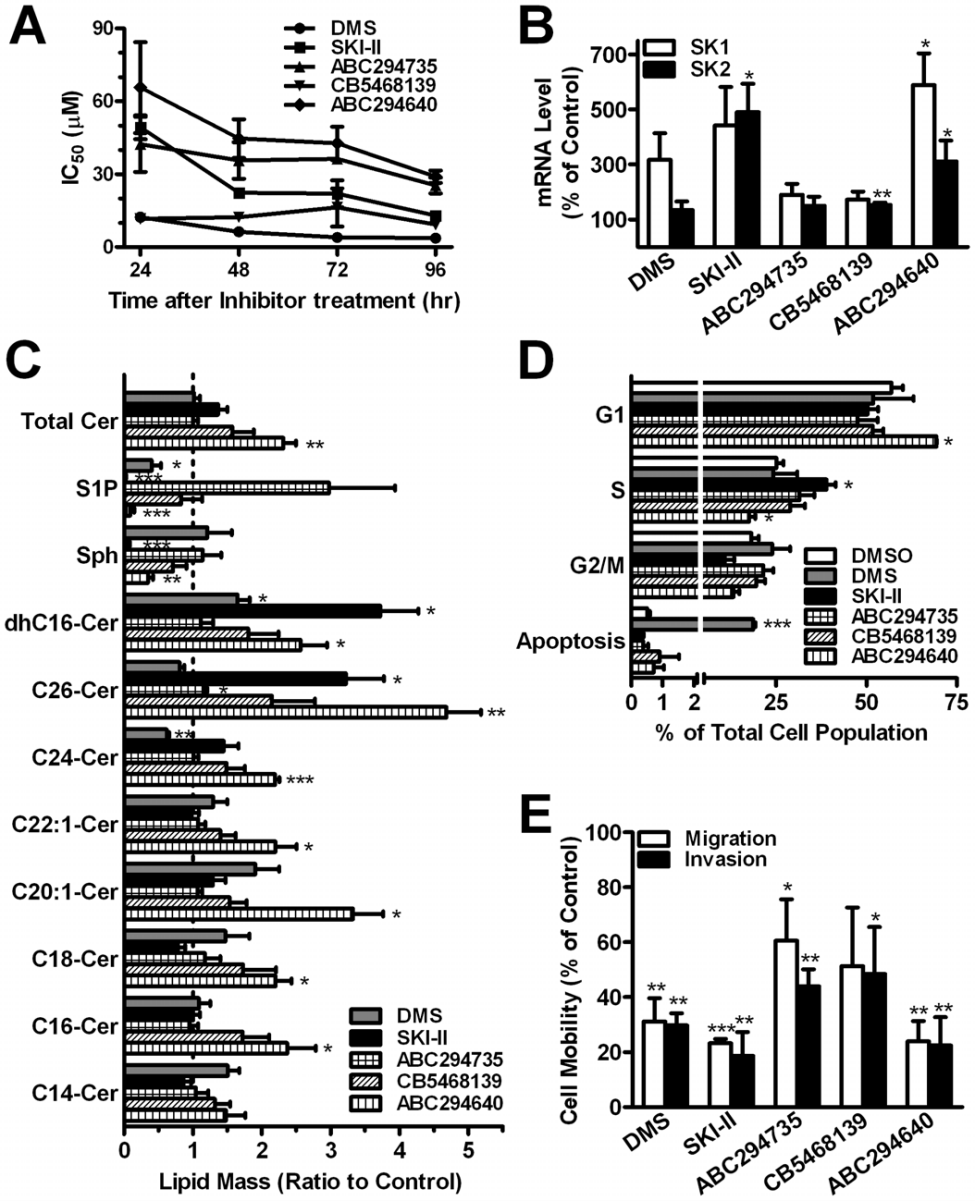
Biochemical and phenotypic effects of SK inhibitors. ***A*** *)* Cytotoxicity - After treatment with varying concentrations of the SK inhibitors for the indicated time periods, cell numbers were quantified. The IC_50_ represents the concentration of the test compound that reduces cell number by 50% compared with DMSO-treated control cultures. In subsequent experiments, cells were incubated with each SK inhibitor at its IC_50_s (DMS - 5 µM, SKI-II - 20 µM, ABC294735 - 35 µM, CB5468139 - 10 µM and ABC294640 - 40 µM) for 48 hours. ***B*** *)* SK expression - After SK inhibitor treatment, mRNAs for SK1 (open bars) and SK2 (filled bars) compared to the vehicle controls (DMSO-treated cells) were calculated. ***C*** *)* Sphingolipid profiling – After SK inhibitor treatment, cells were harvested and sphingolipids were analyzed by mass spectrometric. The bars represent the ratio of the amount of the indicated lipids in drug treated cells compared with control cells. Abbreviations are: C_26_-ceramide (C26-Cer), C_24_-ceramide (C24-Cer), C_20_-ceramide (C20-Cer), C_18_-ceramide (C18-Cer), C_16_-ceramide (C16-Cer), sphingosine (Sph) and dihydrosphingosine (dhSph). ***D*** *)* Cell cycle distribution – After SK inhibitor treatment, cells were harvested and analyzed by flow cytometry. The bars indicate the percentage of cells in each of the indicated cell cycle phases. ***E*** *)* Cell migration and invasion – After SK inhibitor treatment, cells were harvested and migration, through unmodified filters (open bars) and invasion through Matrigel-coated filters (filled bars), were analyzed. The bars indicate the percentage of cells that had migrated compared to control cells. Data are the mean ± SEM of three independent experiments. *p<0.05, **p<0.01, ***p<0.001 versus control.

We previously used A498 kidney adenocarcinoma cells to examine the anticancer effects of selective ablation of SK1 and/or SK2 using siRNAs [17], so the effects of pharmacological inhibition of SK1 and/or SK2 on the proliferation of these cells were determined. All five SK inhibitors reduced the proliferation of A498 cells in a time-dependent manner (Figure 5A). Because DMS is much more potent for inhibiting cell proliferation than it is for inhibiting either SK1 or SK2, its cytotoxic effects are likely mediated by inhibition of other targets. The cytotoxicity and K_i_s for SKI-II are reasonably close, indicating much greater selective targeting to the SKs. Similarly, the potency of ABC294640 toward SK2 is slightly higher than for inhibition of proliferation which may reflect incomplete penetration into the cells. The other phenyladamantane compound ABC294735 demonstrated even a larger multiple for inhibition of cell proliferation in spite of potently inhibiting both SK1 and SK2. Interestingly, cell proliferation was inhibited immediately by the SK1-selective inhibitor CB5468139; however, the IC_50_ was much higher than its K_i_. This is consistent with our previous demonstration that selective ablation of SK1 has a lower effect on proliferation than does ablation of SK2 [17]. For all of the following experiments, cells were treated with the respective IC_50_ for each of the SK inhibitors.

#### SK expression

We previously demonstrated that knockdown of SK2 expression results in overexpression of SK1 in several cell lines [17]. Therefore, the levels of mRNAs for SK1 and SK2 were determined following treatment with each of the SK inhibitors for 48 hr. As shown in Figure 5B, although DMS, SKI-II and ABC294735 are all SK1/2-dual inhibitors, their effects on SK1 and SK2 mRNA expression vary. Treatment with DMS tripled the levels of SK1 mRNA, but only slightly increased SK2 expression. Conversely, treatment with SKI-II increased mRNAs for both SK1 and SK2 by ∼4-fold, which may indicate an attempt to compensate for inhibition of both SK1 and SK2. In the case of SK1, proteosomal degradation of the protein caused by SKI-II might trigger the increase of mRNA to compensate [42], [43]. Neither ABC294735 nor CB5468139 substantially altered the expression of message for either SK1 or SK2. ABC294640-treatment strongly increased SK2 mRNA levels suggesting attempted compensation. Also, consistent with the results of SK2-knockdown in A498 cells [17], inhibition of SK2 activity by ABC294640 dramatically increased SK1 mRNA expression by approximately 5-fold.

#### Sphingolipid metabolism

Because the conversion of sphingosine to S1P by SK is only one step in the dynamic metabolism of sphingolipids [44], an altered flux through SKs may modulate the levels of several sphingolipids. Therefore, we examined the effects of the SK inhibitors on sphingolipids by mass spectrometry (Figure 5C). Treatment with DMS had little impact on the levels of most ceramides, supporting the hypothesis that its effects are mediated by other target proteins. As expected, the SK1/2-dual inhibitor (SKI-II) and SK1-selective inhibitor (CB5468139) increased the total ceramide levels by 30–60%. Treatment with ABC294640 resulted in the greatest increase in total ceramides, reaching 2-times the level of control cells, with significant increases in C_26_-ceramide, C_24_-ceramide, C_22∶1_-ceramide, C_20∶1_-ceramide, C_18_-ceramide and C_16_-ceramide. The levels of sphingosine were dramatically decreased after treatment with SKI-II or ABC294640. Most importantly, compared to the control group, the levels of intracellular S1P were decreased by over 90% after treatment with SKI-II or ABC294640, while treatment of CB5468139 only decreased S1P by less than 20%, which is similar to the amount of S1P remaining after SK2-selective inhibition by ABC294640. Therefore the cell-based analyses of the sphingolipid profiles support the selectivity of these compounds determined in the *in vitro* assays.

#### Cell Cycle and migration/invasion

We previously reported that knockdown of either SK1 or SK2 by siRNA promotes cell cycle arrest, but does not induce apoptosis in A498 cells [17]. Treatment of the cells with DMS did not impact the cell cycle, but drove approximately 20% of the cells into apoptosis (Figure 5D). Given that DMS inhibits PKC activity, this apoptotic response is consistent with other studies [22], [45]. Interestingly, SKI-II arrested cells in S phase with a concomitant decrease in the G2/M phase. ABC294735 and CB5468139 had no significant affect on the percentage of cells in G1, G2/M or apoptosis, but they did slightly increase the S phase percentage. In contrast, ABC294640 showed a distinct G1 arrest with significant decreases in both S and G2/M phases, similar to changes observed upon genetic ablation of SK2. Taken together, the data show that A498 cells respond differently to the SK2-selective inhibitor than they do to SK1-selective or SK1/2-dual inhibitors.

Because S1P is involved in cell migration [46], we investigated the effects of the SK inhibitors on serum-induced migration and invasion (through Matrigel). As shown in Figure 5E, both migration and invasion were more strongly attenuated by treatment with an SK2 inhibitor, either selective (ABC294640) or non-selective (SKI-II), than by the SK1-selective inhibitor (CB5468139).

#### Signaling pathways

**Figure 6:**
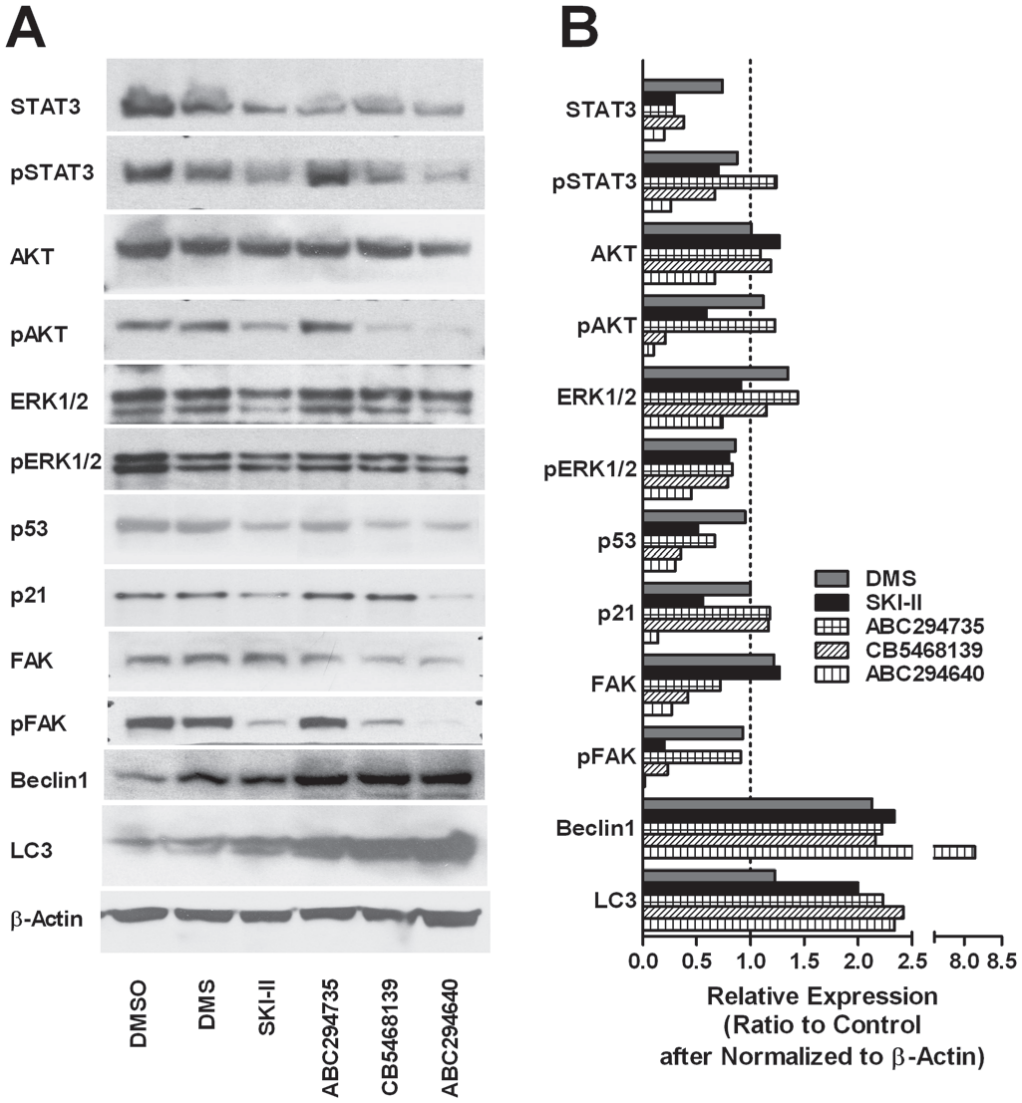
Effects of SK inhibitors on signaling proteins. ***A*** *)* Cells were incubated with the indicated SK inhibitors at their respective IC_50_s for 48 hours and the levels of the indicated proteins were estimated by western blotting. Figures are representative blots of at least three independent experiments. ***B*** *)* Immunoblotting was conducted with the indicated antibodies, and densitometric quantification was done using Image J. Relative expression is represented as the ratio to control after normalization to β-actin.

Effects of the SK inhibitors on markers of proliferation and migration were also assessed. We first determined the expression and activation of STAT3, AKT and ERK, all major regulators of cell proliferation (Figure 6). For STAT3, ABC294640 caused the largest decrease in expression (STAT3) and activation (pSTAT3), with no further decrease occurring in the SK1/2-dual inhibitor-treated groups. None of the inhibitors impacted total AKT expression; however, pAKT was decreased dramatically by treatment with ABC294640, and to a lesser degree by CB5468139 and SKI-II. The pattern of effects on ERK expression was not as clear-cut, but decreases of ERK2 were observed after SKI-II or ABC294640 treatment, as were levels of pERK1/2 after treatment of any of the inhibitors. This is consistent with data from SK2-selective ablation in which there was a greater decrease in ERK2 than ERK1 [17]. Taken together, the data suggest that ABC294640 has a greater impact than the other SK inhibitors on regulating the expression and phosphorylation of STAT3, AKT and ERK.

To assess the mechanism of the observed cell cycle arrest, levels of p53 and p21 were also measured after treatment with the SK inhibitors. Similar to the changes observed with the proliferation markers, SKI-II- or ABC294640-treatment decreased the protein levels of p53 and p21, while CB5468139 reduced the expression of p53, but not p21. DMS had essentially no affect on these proteins, consistent with its lack of affect on cell cycle distribution.

Interestingly, cell migration-related pFAK(Y397) was strongly down-regulated following treatment with SKI-II, ABC294640 or CB5468139. Consistent with inhibition of migration and invasion, these changes were greatest in cells treated with SK2-selective ABC294640. Interestingly, FAK expression and phosphorylation were not altered by DMS, once again indicating that the cellular effects of this compound are markedly different than those of other SK inhibitors. Because we have previously demonstrated that ABC294640 induces autophagy in A498 cells [47], we assessed the effects of the SK inhibitors on beclin1 and LC3. Each of the SK inhibitors elevated the expression of both of these autophagy markers, with ABC294640 having the most pronounced increases.

## Discussion

The SKs are becoming increasingly recognized as potential new targets for anticancer drugs; however, the literature provides differing views on the relative importance of SK1 and SK2 in cancer biology. Therefore, it is critical to define the specific roles as well as the “drugability” of the two SK isoenzymes. We previously used siRNAs to selectively deplete SK1 and/or SK2 from cancer cells, and demonstrated that ablation of SK2 results in stronger anticancer effects than does ablation of SK1 [17]. Additionally, that previous work showed that SK1 cannot restore proliferation, migration or invasion activity to cells that lack SK2 activity [17]. The goal of the present study was to use SK inhibitors to determine if selective pharmacologic inhibition of SK1 and/or SK2 activity replicates the findings of the genetic ablation approach. In studies described herein, we show clear differences in the catalytic rates, substrate affinities and structural topologies for SK1 and SK2. Computational modeling suggests that the nucleotide binding site is highly conserved, whereas the lipid binding sites are divergent between SK1 and SK2. Here, we provide the first comprehensive, side-by-side comparisons of five small molecule SK inhibitors. Each compound was classified as a dual or SK1- or SK2-selective inhibitor, and then the inhibitors were used as pharmacologic probes for several biochemical pathways and cell phenotypes.

It is likely that small molecule inhibitors (SMIs) of the SKs will have advantages over other classes of S1P signaling inhibitors such as monoclonal antibodies. For example, SMIs are more structurally stable, have optimal hydrophobicity to pass through biological membranes to reach the target and are less likely to have immune system tolerance issues. Additionally, many SMIs are orally bioavailable, which simplifies the administration and drug formulation systems. On the other hand, SMI may have lower selectivity than antibodies, and in fact we confirm herein that DMS is likely to exert its cellular effects through targets other than or in addition to SKs. CB5468139, described here for the first time, provides an important indication that SK1-selective agents can be developed, but itself is likely to be too non-selective for development. In contrast, the data presented here support the hypothesis that selective targeting of SK2 by ABC294640 has excellent potential for use in cancer chemotherapy [34], [36], [48]. Importantly, we now show that ABC294640 has similar or greater potency for inhibiting cancer cell proliferation and migration compared to SK1/2-dual and SK1-selective inhibitors. This indicates that the functions of SK2-generated S1P cannot be fully compensated by SK1-generated S1P, possibly due to their different subcellular localizations [17], [49], [50]. Also, sphingolipid profiling demonstrated significant increases in ceramide species combined with depletion of S1P after ABC294640 treatment which likely intensifies its anti-proliferative activity. Interestingly, the significant increase of SK2 expression in response to exposure to ABC294640 may also contribute to its anti-cancer activity because SK2 possesses a pro-death BH3 domain. On one hand, ABC294640 treatment inhibits the mitogenic kinase function of SK2; while on the other hand, the overexpression of the BH3 domain could provide a magnified pro-death stimulus. This is consistent with studies that showed that overexpression of SK2 by transfection results in apoptosis [15], [51]. Further study of the promoter elements responsible for SK2 transcription would be of considerable interest to elucidate the mechanism for induction by ABC294640.

The expression and phosphorylation of pro-survival signaling proteins such as STAT3, AKT, ERK and FAK were markedly impacted by the SK2-selective inhibitor ABC294640, and to a less degree by other SK inhibitory compounds. ABC29460 also disrupted the cell cycle with arrest in G1 and reduced expression of p53 and p21, which mimicked the selective knockdown of SK2 with siRNA [17]. Flow cytometric analyses did not reveal significant increases in apoptosis after treatment with ABC294640; however elevation of the autophagy markers Beclin1 and LC3 suggest that the cells are dying by excessive autophagy. Although autophagy is recognized as a survival mechanism under most conditions, it is also capable of inducing cell death characterized by extensive digestion of intracellular organelles leading to large numbers of autophagic vacuoles [52], [53]. Furthermore, a number of small molecules (including several anticancer drugs) activate autophagy in cancer cells both *in vivo* and *in vitro* [54]. Among the SK inhibitory compounds tested, the SK1/2-dual inhibitor SKI-II is the only one that had the same degree of anti-proliferative and anti-migratory activity as ABC294640. DMS had less impact; whereas ABC294735 was largely inactive except for the induction of autophagy. Our previous RNA interference studies suggest that selective inhibition of SK1 should result in only mild suppression of cell growth and migration [17]. CB5468139 had relatively strong activity in certain assays including cell proliferation, elevation of LC3 cleavage and inhibition of AKT phosphorylation, but only modest effects on sphingolipid profiles, cell cycle distribution and migration. This disparity is likely due to off-target effects of CB5468139, supporting the hypothesis that compounds targeting the sphingosine binding site rather than the ATP binding site provide more pure pharmacologic probes of SK activity and potentially less toxic therapeutic agents. Additionally, SK2-selective inhibitors may effectively deplete the nuclear S1P pool of tumor cells while having less impact on circulating S1P levels which are important for the maintenance of normal vascular and immune function [55].

Overall, these studies support the continued development of ABC294640, which is currently undergoing Phase 1 clinical testing in patients with advanced solid tumors. Future development of additional SK2-selective inhibitors may provide more effective anticancer agents than SK1/2-dual or SK1-selective inhibitor.

## Supporting Information

Figure S1 **Kinase profiling for CB5468139.** The inhibitory effects of CB5468139 (2 µM) on the indicated protein kinases were tested using KinaseSeeker assay technology (Luceome Biotech. LLC). The bars indicate the percentage of kinase activity compared to vehicle (DMSO) control lysates.
(TIF)

Figure S2 **Sequence alignment of DAG Kinase, SK1 and SK2.** Sequences for the three proteins were aligned using BioEdit and the ClustalW algorithm as described in the Materials and Methods section. Note that the amino terminal of SK1 was truncated for the homology model, and the start of the homology models begins and ends at the black brackets.
(TIF)

Figure S3 **DAG Kinase (DAGK) Inhibition.** The activity of recombinant DAGK (Enzo Life Sciences) was measured under initial velocity conditions using the ADP-Quest system described in the Materials and Methods section in the presence of the indicated concentrations of DMS (•), SKI-II (▪), ABC294735 (▴), CB5468139 (⧫) or ABC294640 (▾). Data are mean ± SD of triplicates of a representative of three independent experiments.
(TIF)
