# Supplementary figures and images for: Characterization of Isoenzyme-Selective Inhibitors of Human Sphingosine Kinases
Source: PLoS One. 2012 Sep 10;7(9):e44543. doi: 10.1371/journal.pone.0044543 (PMC3438171; doi:10.1371/journal.pone.0044543)

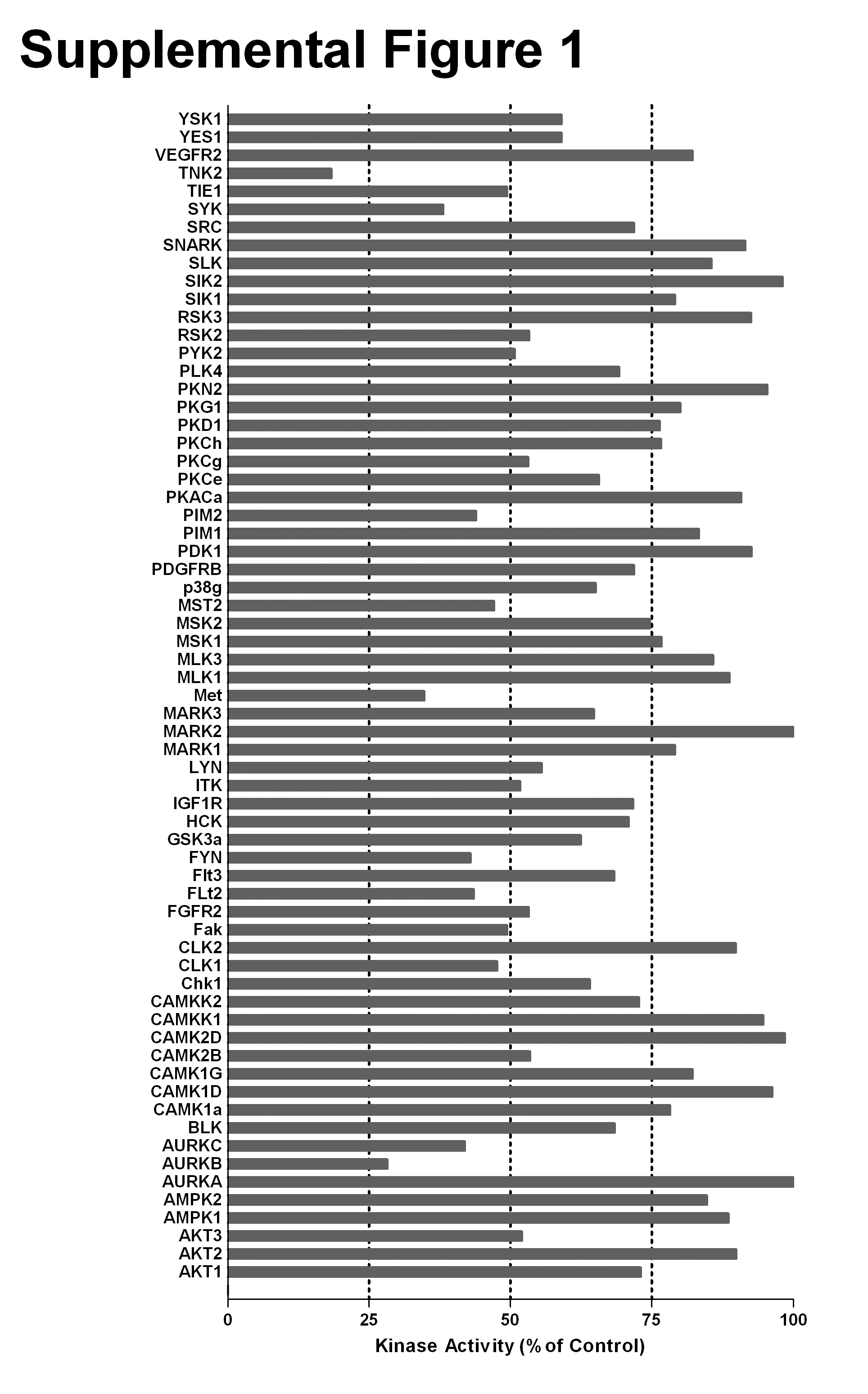

Supplement: Figure S1 — Kinase profiling for CB5468139. The inhibitory effects of CB5468139 (2 µM) on the indicated protein kinases were tested using KinaseSeeker assay technology (Luceome Biotech. LLC). The bars indicate the percentage of kinase activity compared to vehicle (DMSO) control lysates. (TIF) [file pone.0044543.s001.tif]

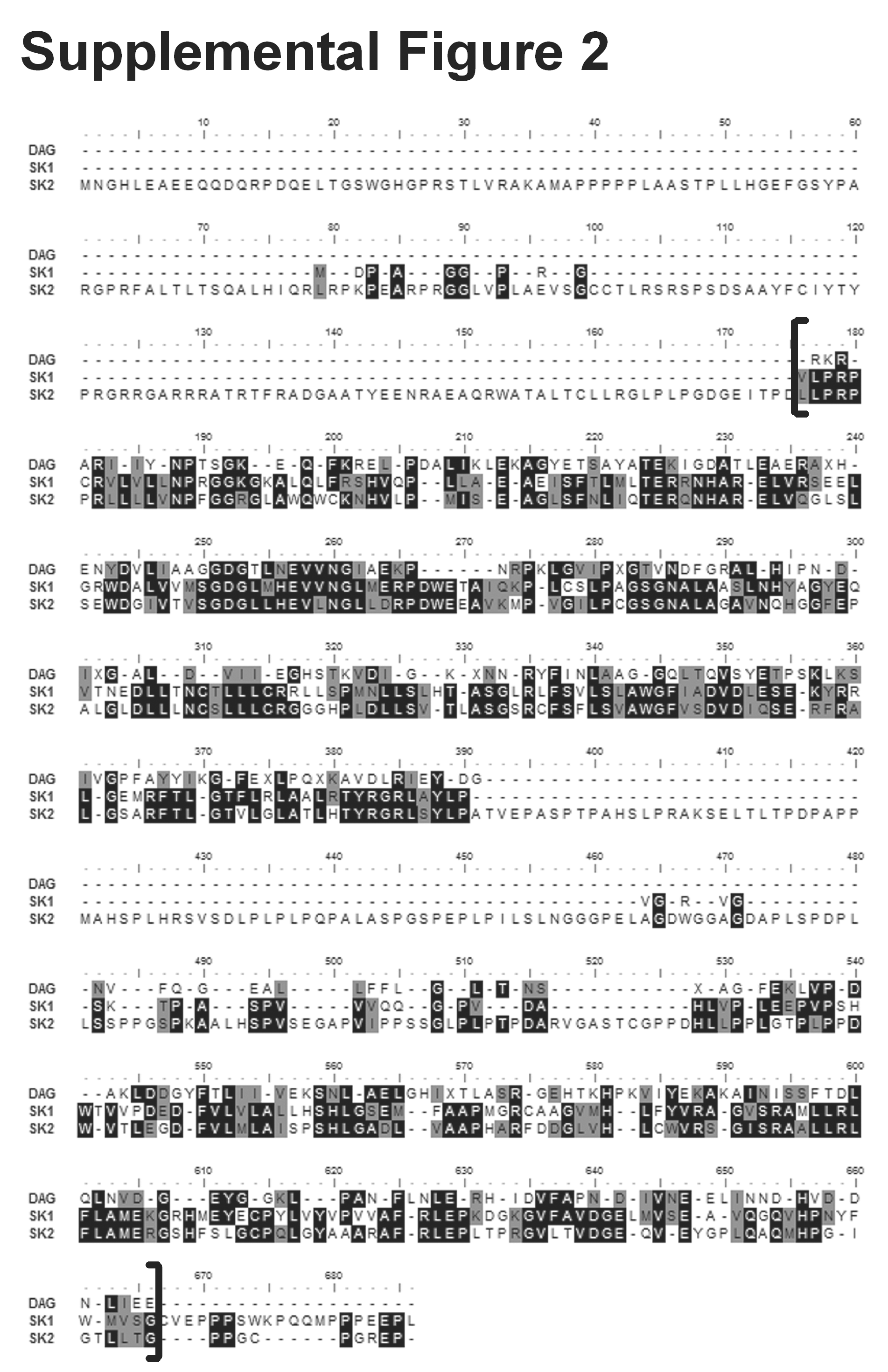

Supplement: Figure S2 — Sequence alignment of DAG Kinase, SK1 and SK2. Sequences for the three proteins were aligned using BioEdit and the ClustalW algorithm as described in the Materials and Methods section. Note that the amino terminal of SK1 was truncated for the homology model, and the start of the homology models begins and ends at the black brackets. (TIF) [file pone.0044543.s002.tif]

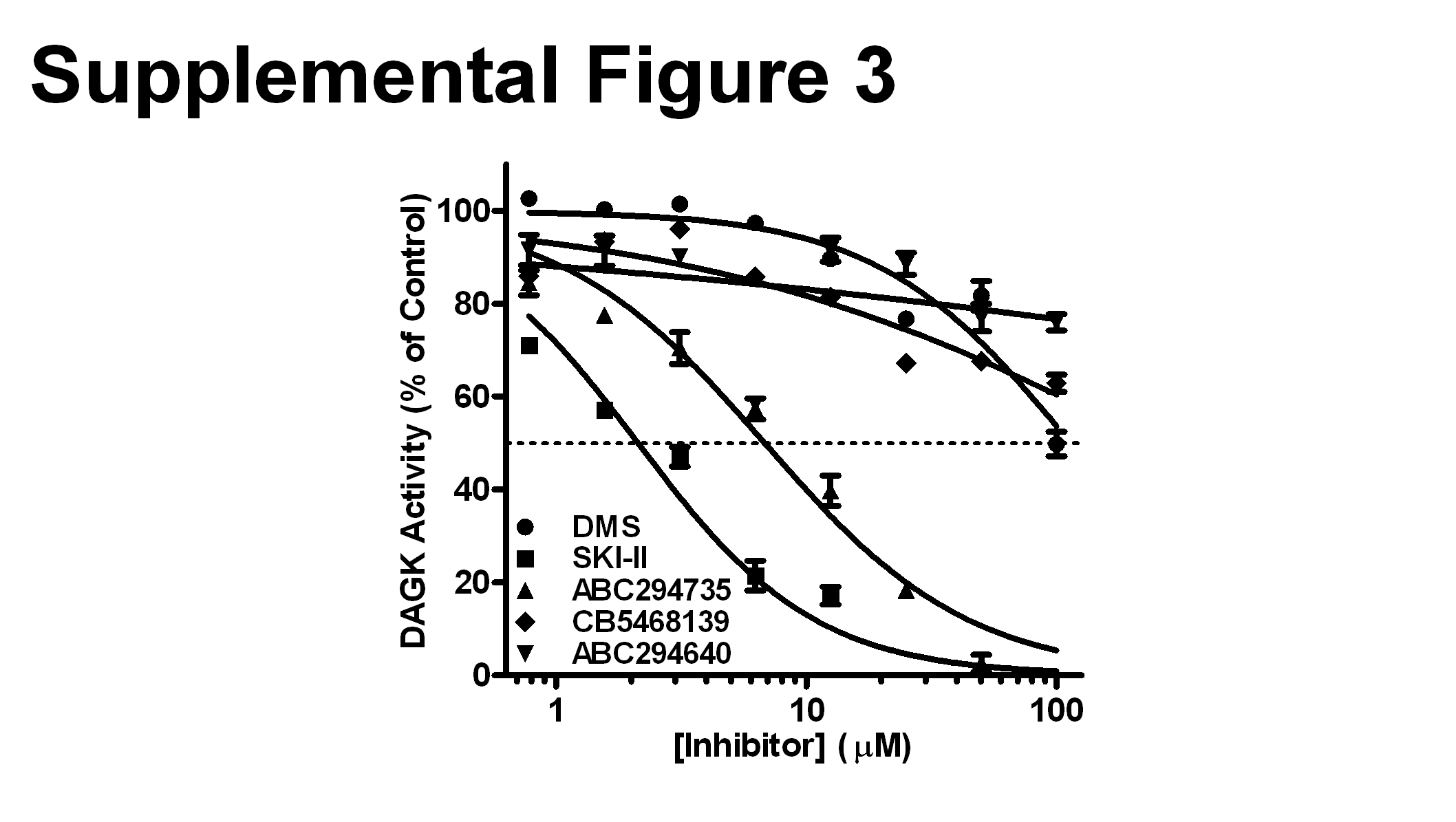

Supplement: Figure S3 — DAG Kinase (DAGK) Inhibition. The activity of recombinant DAGK (Enzo Life Sciences) was measured under initial velocity conditions using the ADP-Quest system described in the Materials and Methods section in the presence of the indicated concentrations of DMS (•), SKI-II (▪), ABC294735 (▴), CB5468139 (⧫) or ABC294640 (▾). Data are mean ± SD of triplicates of a representative of three independent experiments. (TIF) [file pone.0044543.s003.tif]
